# Status of Measles Elimination in Eleven Countries with High Routine Immunisation Coverage in The WHO African Region

**Published:** 2018-07-28

**Authors:** Balcha Masresha, Richard Luce, Messeret Shibeshi, Reggis Katsande, Amadou Fall, Joseph Okeibunor, Goitom Weldegebriel, Richard Mihigo

**Affiliations:** 1 WHO Regional Office for Africa, Brazzaville, Congo; 2 WHO Inter-country Support Team for Central Africa, Libreville, Gabon; 3 WHO Inter-country Support Team for East and Southern Africa, Harare, Zimbabwe; 4 WHO Inter-country Support Team for Western Africa, Ouagadougou, Burkina Faso

**Keywords:** Measles Elimination, Immunization, Coverage, Status, WHO African Region

## Abstract

**Background:**

Measles elimination is defined as the absence of endemic measles virus transmission in a defined geographic area for at least 12 months in the presence of a well-performing surveillance system. The WHO framework for verification of measles elimination indicates that the achievement of measles and/or rubella elimination should be verified for individual countries.

**Objective:**

We identified 11 high performing countries based on their first dose measles vaccination coverage, and looked at their performance across the various programmatic parameters, to see if they are ready to undertake the verification of measles elimination.

**Methods:**

We identified 11 countries with >90% measles first dose coverage for the most recent 5 years according to the WHO UNICEF estimates of national immunisation coverage. We analysed vaccination coverage and surveillance performance in these countries.

**Results:**

Algeria, Botswana, Gambia, Mauritius, Rwanda, Seychelles have maintained measles first dose (MCV1) coverage of 95% or more since 2011. In 2015, only Algeria, Cape Verde and Seychelles had coverage of 95% or more for the second dose of measles vaccine (MCV2). Of the 22 supplemental immunisation activities (SIAs) among the 11 countries, only 6 had administrative coverage of less than 95%. Only Rwanda and Lesotho attained the case-based surveillance performance targets in all the five years.

**Conclusion:**

Despite their high routine first dose measles immunisation coverage, all of the 11 countries have some program gaps indicating that they do not meet all the criteria to undergo verification of elimination at this point. It is recommended for these countries to set up national verification committees as per the WHO framework for verification of measles elimination, in order to initiate the documentation and monitoring of progress, and to address programmatic gaps in the coming years.

## Introduction

The attainment of sustained high population immunity through high levels of measles vaccination coverage in the routine immunisation system is the backbone of measles elimination efforts. Equally important is the presence of a sensitive surveillance system that can detect and confirm suspected measles cases, and guide the immunisation program towards swift action to limit the spread of disease. Previous studies have investigated the relationship between vaccination coverage and the incidence of measles^1^, ^2^.

Countries in the WHO African Region have been engaged in measles mortality reduction activities since 2011 by implementing WHO recommended strategies^3^. In 2011, the Member States of the WHO African Region adopted a measles elimination goal to be reached by the end of 2020 with the following targets: Measles incidence of less than 1 case per million population at national level; at least 95% measles immunization coverage at national level and in all districts; minimum 95% coverage in all measles SIAs; at least 80% of districts investigating one or more suspected measles cases within a year; and a non-measles febrile rash illness rate of at least 2 per 100 000 population at national level^4^. Through the implementation of these strategies, the Region has documented 85% reduction in estimated measles deaths between 2000 and 2015 5.

Measles elimination is defined as the absence of endemic measles virus transmission in a defined geographic area (e.g., region or country) for ≥ 12 months in the presence of a well-performing surveillance system. The attainment of measles elimination needs to be documented and independently verified in a structured manner according to the WHO framework for verification of measles elimination. The framework indicates that the achievement of measles and/or rubella elimination should be verified following a standardised process. In addition to the eventual verification of elimination, the process is expected to help countries to monitor progress towards elimination^6^.

This article looks at the overall level of implementation of measles elimination strategies in countries that have maintained more than 90% first dose measles vaccination coverage (MCV1) for the most recent 5 years for which data is available, and their readiness for verification of elimination.

## Methods

WHO and the United Nations Children’s Fund (UNICEF) use data from administrative records reported annually by Member States and from surveys to produce national coverage estimates with first dose measles (MCV1) and the second dose of measles (MCV2) through routine immunization services^7^. Supplemental immunization activities (SIAs) generally are carried as initial, nationwide catch-up SIA targeting all children aged 9 months–14 years, with the goal of eliminating susceptibility to measles in the childhood population; and as periodic follow-up SIAs targeting all children born since the last SIA. Follow-up SIAs are often conducted nationwide every 2–4 years, and focus on children aged 9–59 months, in order to eliminate any measles susceptibility in recent birth cohorts and to protect children who did not sero-convert following the receipt of routine doses of measles vaccine. The target age range for follow-up SIAs may be widened to include older children based on the measles susceptibility pattern in countries.

SIA administrative coverage at the subnational and national level is calculated by tallying the number of administered doses and dividing by the target population. Target population figures are often projections from census data. Countries often conduct post-SIA coverage surveys to validate the administrative vaccination coverage achieved during the SIA^8^.

Countries report the number of measles cases annually to WHO and UNICEF using data either case-based or aggregate surveillance systems. Effective measles surveillance includes case-based surveillance with laboratory testing to confirm cases. In case-based surveillance system, each suspected measles case is investigated. A suspected measles case is defined as a case of generalized maculo-papular rash and fever plus one of the following: cough, runny nose, or conjunctivitis. For each suspected measles case, an individual case investigation form is completed and a blood specimen collected and sent to the national laboratory for testing for measles-specific immunoglobulin M (IgM) antibody^9^.

The surveillance performance of is regularly monitored using standard indicators which look at the sensitivity of the case detection, and also the geographic spread of case notification and investigation. Among the indicators in use, two are considered as the principal monitoring indicators: Non-measles febrile rash illness rate (target of at least 2 per 100 000 population) and the proportion of districts that have investigated at least one suspected case of measles with blood specimen per year (target of 80% or more per year).

We analysed the WHO UNICEF estimates of national immunisation coverage (WUENIC) data for the first measles vaccination dose (MCV1) and identified 11 countries that have maintained more than 90% MCV1 coverage for the most recent 5 years for which data is available (2011 – 2015). Subsequent analysis focused on these 11 countries, and included analysis of MCV2 coverage, as well as the coverage data from supplemental immunisation activities (SIAs) conducted in the last 10 years (2007 – 2016). We also reviewed the case-based surveillance data and surveillance performance from the countries for the most recent 5 years period (2012 – 2016).

## Results

We identified 11 countries (Algeria, Botswana, Burundi, Cape Verde, Gambia, Lesotho, Mauritius, Rwanda, Sao Tome and Principe, Seychelles and Tanzania) that had MCV1 coverage of at least 90% (according to the WUENIC) in the 5 years between 2011 and 2015. Of these countries, 9 countries give MCV1 at 9 months of age, while Mauritius has a 12 months schedule and Seychelles provides MCV1 at 15 months of age. All of these countries have MCV2 in their routine immunisation schedule, provided in the second year of life in 8 countries. Algeria and Seychelles give it at 6 years of age, and Mauritius has a 5 years schedule. As of May 2017, Algeria, Burundi and Lesotho use monovalent measles vaccine while the other countries use Measles-Rubella (Botswana, Rwanda, Gambia, Tanzania, Sao Tome and Principe) or Measles-Mumps-Rubella vaccine (Cape Verde, Mauritius and Seychelles).

According to the WUENIC, 7 out of the 11 countries have coverage of 95% or more in 2015. Algeria, Botswana, Gambia, Mauritius, Rwanda, Seychelles have maintained coverage of 95% or more since 2011. (Table 1) The average MCV1 coverage between 2006 – 2015 range from 91% in Sao Tome and Principe to 99% in Mauritius.

Six of the 11 countries introduced MCV2 within the past 5 years. In 2015, only Algeria, Cape Verde and Seychelles had coverage of 95% or more for MCV2. Sao Tome & Principe and Tanzania started reporting MCV2 since 2014, and Rwanda since 2015. The drop-out rates between MCV1 and MCV2 in 2015 is more than 20% in Burundi, Gambia and Tanzania, and between 10 and 19% in Botswana, Mauritius, and Sao Tome and Principe (Table 1).

The measles or measles-rubella (MR) SIAs conducted in the last 10 years in these countries are tabulated in Table 2. Of the 22 SIAs among the 11 countries, only 6 had administrative coverage of less than 95%. Coverage surveys were done in only 5 of the SIAs. Algeria, Mauritius and Seychelles did not do any nationwide measles or measles-rubella SIAs (Table 2).

As of 2016, these 11 countries, with the exception of Sao Tome and Principe, each have a national measles serological confirmatory laboratory that is part of the African Regional measles-rubella laboratory network. Mauritius, Sao Tome and Principe, and Seychelles have health facility based surveillance systems which include notification of measles cases, but do not do case based surveillance for measles and rubella using the WHO AFRO protocol. The countries report cases to WHO and UNICEF through the annual WHO-UNICEF joint reporting form (JRF reporting tool), and do not generate indicators for measles case based surveillance performance monitoring as per the WHO AFRO protocol. As of the end of 2016, Rwanda and Lesotho are the only two countries that have attained the performance targets for the two principal measles surveillance monitoring indicators in all the five years, while Botswana and the Gambia have met both targets for at least 4 of the 5 years between 2011 - 2015. In 2015, three of the 8 countries had non measles febrile rash illness rates in excess of 4 per million population. Two countries had similarly high rates in 2016 (Table 3).

The incidence of confirmed measles (confirmed by lab, epidemiological linkage and clinically) is displayed in Table 4. Gambia and Lesotho had incidence levels of more than 5 confirmed cases per million population in 2016, while Botswana, Cape Verde and Tanzania have attained the elimination target of < 1 confirmed measles case per million (Table 4).

## Discussion

These 11 countries have maintained very high MCV1 coverage for at least 5 years, and have all introduced MCV2 in the routine immunisation schedule. With the exception of Algeria and Seychelles, the MCV2 coverage remains below the levels required to attain herd immunity and maintain measles elimination. Eight countries have been conducting periodic measles SIAs to close immunity gaps. However, not all countries have been conducting post-campaign coverage surveys to validate administrative coverage.

These 11 countries have maintained low incidence of measles as compared to the Regional average^10^. However, Gambia, Lesotho and Rwanda reported incidence rates of 5 or more per million population in 2016. The quality of case-based measles surveillance has been consistently below the targets in Algeria, Burundi, Cape Verde and Tanzania.

The experience from various countries shows that to bring down and sustain the incidence at less than 1 per million population and undertake verification of elimination, countries will need to maintain high population immunity to measles; increase the sensitivity of the measles surveillance system, including the characterisation of measles virus genotypes^11^–^13^. Moreover, all countries should develop plans to be able to detect and rapidly respond to measles importations, which can easily occur across international borders. For example, Rwanda, Burundi and Tanzania share borders with DR Congo, which has been experiencing large measles outbreaks over the past years^10^, ^14^–^15^. Even after a significant reduction of measles incidence, countries may experience resurgence or importations of measles virus which requires that sensitive disease surveillance and monitoring of circulating viral genotype be in place beforehand in order to detect, document and respond to these situations^16^–^18^.

Mauritius, Sao Tome and Principe, and Seychelles have not yet established case-based surveillance for measles, but they report aggregate number of cases to WHO and UNICEF through the annual joint reporting tool. Moreover, they do not generate indicators for measles surveillance quality as per the WHO protocol. These countries should include the core variables into their systems in order to be able to monitor the quality of surveillance, measure their performance using the standard indicators, and generate evidence for the verification of measles elimination.

The WHO framework for the verification of measles elimination requires that countries generate and document programmatic evidence related to population immunity, surveillance quality and disease incidence. In addition, the lines of evidence required for the verification exercise include vaccination program sustainability, and genotyping data^6^.

Despite the high routine immunisation coverage, the eleven countries have some program gaps and do not meet all the criteria for the verification of elimination. However, these countries should set up national verification committees as per the WHO protocol in order to initiate the documentation and monitoring of progress, and to address programmatic gaps in the coming years.

There are limitations to this analysis. The coverage figures quoted in this report are from 2015. The WHO UNICEF national coverage estimates for 2016 are not yet available. The reported burden of measles may be underestimated, because not all persons with suspected measles seek care and not all of those who seek care are reported. Data from aggregate reporting systems was not included in this analysis, except for the reports from Mauritius, Seychelles and Sao Tome and Principe. The performance of the national measles laboratories is not included in this analysis.

## Figures and Tables

**Table 1:** MCV1 and MCV2 coverage. 2011 - 2015. WHO UNICEF coverage estimates.

	MCV1 coverage					MCV2 coverage				
	2011	2012	2013	2014	2015	2011	2012	2013	2014	2015
Algeria	95%	95%	95%	95%	95%	96%	95%	93%	99%	99%
Botswana	97%	97%	97%	97%	97%	-	-	83%	85%	85%
Burundi	93%	93%	98%	94%	93%	-	-	51%	60%	65%
Cape Verde	96%	91%	91%	93%	92%	99%	94%	89%	79%	95%
Gambia	91%	95%	96%	96%	97%	-	56%	53%	73%	77%
Lesotho	92%	91%	90%	90%	90%	82%	82%	82%	82%	82%
Mauritius	99%	99%	99%	98%	99%	89%	90%	85%	85%	85%
Rwanda	95%	97%	95%	97%	97%	-	-	-	-	87%
Sao Tome & Principe	91%	92%	91%	92%	93%	-	-	-	71%	76%
Seychelles	99%	98%	97%	99%	98%	99%	99%	97%	98%	98%
Tanzania	93%	97%	99%	99%	99%	-	-	-	29%	57%

**Table 2:** Timing and coverage of Measles/ Measles –rubella SIAs. (2007 - 2016)

COUNTRY	Year	Type of SIAs	Age group of children targeted	Number of children vaccinated	Coverage (percent of target)	% districts with 95% or above coverage	coverage survey result
Botswana	2009	Measles Follow-up	9 - 59 months	187,380	111%		
	2013	Measles Follow-up	9 - 59 months	198,341	95%	54.0%	
	2016	MR catch up	9 mo - 14 yrs	674,150	95%	66.7%	
Burundi	2009	Measles Follow-up	9 - 59 months	1,321,915	95%		
	2010	outbreak response	9 mo - 14 years	446,775	94%		
	2012	Measles Follow-up	9 - 59 months	1,459,304	103%	82.2%	
Cape Verde	2009	Measles Follow-up	9 - 59 months	47,667	87%		
	2013	MR catch-up	9 mo – 24 yrs	240,166	95%	45.5%	
Gambia	2007	Measles Follow-up	9 - 59 months	241,214	96%		
	2011	Measles Follow-up	9 - 59 months	294,579	95%	52.5%	
	2016	MR catch up	9 mo - 14 yrs	779,654	97%	85.7%	
Lesotho	2007	Measles Follow-up	9 - 59 months	196,490	92%		
	2010	Measles Follow-up	6 mo - 14 years	558,335	91%	40.0%	94.3%
	2013	Measles Follow-up	9 - 59 months	147,676	72.7%	90.0%	92.0%
Rwanda	2009	Measles Follow-up	9 - 59 months	1,350,125	101%		
	2013	MR catch-up	9 mo - 14 years	4,391,081	103%	90.0%	98.0%
Saotome & Principe	2007	Measles catch-up	9 mo - 14 years	64,487	101%		
	2012	Measles Follow-up	9 - 59 months	22,528	105.4%	100.0%	
	2016	MR catch up	9 mo - 14 yrs	77,285	107%	100.0%	
Tanzania	2008	Measles Follow-up	9 - 59 months	10,826,519	86%		
	2011	Measles Follow-up	9 - 59 months	6,686,663	97%	60.0%	91.6%
	2014	MR catch-up	9 mo - 14 yrs	20,529,629	97%	59.3%	89.0%

**Table 3:** Measles surveillance performance 2012 - 2016.

	Non-measles Febrile Rash Illness Rate per 100,000 population by year (target ≥ 2:100,000)					Proportion of districts reporting at least 1 case with blood specimen by year (target ≥ 80%)				
	2012	2013	2014	2015	2016	2012	2013	2014	2015	2016
Algeria	0.1	0.2	0.1	0.1	0.1	38%	31%	40%	42%	42%
Botswana	4	14.9	39	6.9	6.3	72%	88%	96%	84%	84%
Burundi	0.6	0.4	1.8	1.3	1.3	66%	36%	64%	69%	69%
Cape Verde	0.2	0	0	0	0	6%	6%	0%	0%	6%
Gambia	4.4	7.5	5.3	4	0.9	100%	83%	100%	78%	56%
Lesotho	9.4	20.4	27	14.1	3.2	90%	100%	100%	90%	90%
Rwanda	6.5	6.4	3.7	3.6	7.3	100%	100%	94%	94%	94%
Tanzania	1.2	0.1	1.7	0.8	1.9	94%	81%	100%	99%	100%

**Table 4:** Number and incidence of confirmed measles cases. 2012 - 2016.

	Total population in millions as of 2016	Total number of confirmed measles cases					Incidence rate of confirmed measles per million population				
		2012	2013	2014	2015	2016	2012	2013	2014	2015	2016
Algeria	39.5	6	0	0	62	46	0.2	1.6	0	1.6	1.2
Botswana	2.1	4	1	88	2	1	2	1	41.1	1	0.5
Burundi	9.6	49	0	5	9	13	5.5	0.9	0.5	0.9	1.4
Cape Verde	0.5	0	0	0	0	0	0	0	0	0	0
Gambia	1.9	0	1	2	23	40	0	13.2	1.1	13.2	21.4
Lesotho	1.9	0	1	0	1	13	0	0.5	0	0.5	6.7
Rwanda	11	81	17	5	1	58	7.9	0.1	0.5	0.1	5
Tanzania	50.1	736	21	62	18	36	16.4	0.4	1.3	0.4	0.7
Mauritius*	1.3	0	0	0	-	-					
Seychelles*	0.1	0	0	0	0	-					
Sao tome & Principe*	0.2	0	0	0	0	-					

* Number of cases officially reported to WHO-UNICEF through the JRF.
